# FKBPL is associated with metabolic parameters and is a novel determinant of cardiovascular disease

**DOI:** 10.1038/s41598-020-78676-6

**Published:** 2020-12-10

**Authors:** Andrzej S. Januszewski, Chris J. Watson, Vikki O’Neill, Kenneth McDonald, Mark Ledwidge, Tracy Robson, Alicia J. Jenkins, Anthony C. Keech, Lana McClements

**Affiliations:** 1 NHMRC Clinical Trials Centre, University of Sydney, Sydney, NSW Australia; 2 Wellcome-Wolfson Institute for Experimental Medicine, School of Medicine, Dentistry and Biomedical Science, Queen’s University Belfast, Belfast, UK; 3 Centre for Public Health, School of Medicine, Dentistry and Biomedical Science, Queen’s University Belfast, Belfast, UK; 4 STOP-HF Unit, St. Vincent’s University Hospital, Dublin 4, Ireland; 5 School of Medicine, University College Dublin, Dublin, Ireland; 6 School of Pharmacy and Biomolecular Sciences, Irish Centre for Vascular Biology, RCSI University of Medicine and Health Sciences, Dublin, Ireland; 7 School of Life Sciences, University of Technology Sydney, Sydney, NSW Australia

**Keywords:** Biochemistry, Biomarkers, Cardiology, Diseases, Endocrinology, Molecular medicine

## Abstract

Type 2 diabetes (T2D) is associated with increased risk of cardiovascular disease (CVD). As disturbed angiogenesis and endothelial dysfunction are strongly implicated in T2D and CVD, we aimed to investigate the association between a novel anti-angiogenic protein, FK506-binding protein like (FKBPL), and these diseases. Plasma FKBPL was quantified by ELISA cross-sectionally in 353 adults, consisting of 234 T2D and 119 non–diabetic subjects with/without CVD, matched for age, BMI and gender. FKBPL levels were higher in T2D (adjusted mean: 2.03 ng/ml ± 0.90 SD) vs. non-diabetic subjects (adjusted mean: 1.79 ng/ml ± 0.89 SD, *p* = 0.02), but only after adjustment for CVD status. In T2D, FKBPL was negatively correlated with fasting blood glucose, HbA1c and diastolic blood pressure (DBP), and positively correlated with age, known diabetes duration, waist/hip ratio, urinary albumin/creatinine ratio (ACR) and fasting C-peptide. FKBPL plasma concentrations were increased in the presence of CVD, but only in the non-diabetic group (CVD: 2.02 ng/ml ± 0.75 SD vs. no CVD: 1.68 ng/ml ± 0.79 SD, *p* = 0.02). In non-diabetic subjects, FKBPL was positively correlated with an established biomarker for CVD, B-type Natriuretic Peptide (BNP), and echocardiographic parameters of diastolic dysfunction. FKBPL was a determinant of CVD in the non-diabetic group in addition to age, gender, total-cholesterol and systolic blood pressure (SBP). FKBPL may be a useful anti-angiogenic biomarker in CVD in the absence of diabetes and could represent a novel CVD mechanism.

## Introduction

Diabetes is becoming an epidemic disease of global proportion with over 460 million people living with the condition in 2019; type 2 diabetes mellitus (T2D) accounts for the majority^1^. People with diabetes have up to three-fold higher^2^ incidence of cardiovascular disease (CVD), the leading cause of death globally^3^. Despite several long-term clinical trials, such as UKPDS, showing that glucose lowering strategies are effective in reducing the incidence of CVD in diabetes, this is still significantly higher even in optimally treated patients^4^. Therefore, it is clear that better stratification of patients and more effective personalised treatment strategies are needed. Natriuretic Peptides (NPs), including B-type Natriuretic Peptide (BNP) and N-terminal pro BNP (NT-proBNP), are the most reliable biomarkers for identifying people with heart failure (HF), both in heart failure with preserved ejection fraction (HFpEF) and HF with reduced ejection fraction (HFrEF), previously known as diastolic and systolic HF, respectively^5^. NPs have also been shown to predict future major adverse cardiovascular events in patients with CVD risk factors^6^. Inflammation appears to be one of the main underlying mechanisms leading to CVD in diabetes, whereas in the absence of diabetes other mechanisms are also implicated including angiogenesis, remodelling and metabolism^7^. Identifying novel mechanisms, which can be explored as biomarkers or therapeutic targets of both T2D and/or CVD, is important for the prevention of associated complications.

FK506 binding-protein like (FKBPL) is a novel angiogenesis-related protein, which inhibits endothelial cell migration through disruption of actin/tubulin dynamics via the CD44 pathway^8,9^, and regulates glucocorticoid receptor activity^10^. Recently, FKBPL has also been shown to target inflammatory the STAT3 pathway^11^. A first-in-class pre-clinical candidate peptide, AD-01, based on the active anti-angiogenic domain of FKBPL, was developed and has been extensively tested pre-clinically^8,12,13^. A clinical therapeutic peptide, ALM201, has completed a Phase I clinical trial for the treatment of solid tumours (EudraCT No: 2014-001175-31)^14^. As anticipated, FKBPL haploinsufficient (*Fkbpl*^+*/-*^) mice demonstrated that FKBPL has a critical role in physiological, developmental and pathological angiogenesis; blood vessel development was also impaired when FKBPL was knocked down in zebrafish^15^. Interestingly, homozygous knockout of the FKBPL gene led to embryonic lethality, highlighting its important role in developmental angiogenesis. Notably, *Fkbpl*^+*/−*^ embryos were viable, however they showed signs of early endothelial dysfunction^15^, but developed normally with some level of vascular dysfunction and leakiness^15^.

Vascular and endothelial dysfunction precipitated by hyperglycaemia are well-studied aberrant mechanisms in diabetes and CVD^16–18^. Microangiopathy, as a result of endothelial dysfunction and angiogenic imbalance, is implicated in the development of CVD in diabetes^19^. Therefore, since FKBPL regulates angiogenesis and glucocorticoid receptor signalling, we hypothesised that it could also have a role in the pathogenesis of vascular damage in T2D as well as CVD. The main aims of our study were to investigate the relationship between circulating FKBPL levels and the presence of T2D and/or CVD and determine the correlations with metabolic and cardiac function parameters. This report investigates circulating FKBPL levels in diabetes and/or CVD for the first time.

## Methods

### Subjects and samples

Plasma samples used in this study were collected from 234 subjects with T2D aged 50–75 years as part of the prospective, randomised, double-blind, placebo controlled Fenofibrate Intervention and Event Lowering in Diabetes (FIELD) study, from well-characterised patients who were recruited and screened but not randomised thereafter to receive fenofibrate or placebo. Participants in the FIELD study were statin-naïve and had the following lipid profile: total-cholesterol concentration of 3.0–6.5 mmol/L and a total-cholesterol/HDL-cholesterol ratio of 4.0 or more or plasma triglyceride of 1.0–5.0 mmol/L. Further details on the criteria for the recruitment into the FIELD study were described previously^20^ (ClinicalTrials.gov identifier: ISRCTN64783481). These subjects were matched for age, body mass index (BMI) and gender to non-diabetic controls, at an approximately 2:1 ratio (234 T2D plasma samples vs. 130 non-diabetes plasma samples), drawn from the STOP-HF study^21^ (ClinicalTrials.gov identifier: NCT00921960). The STOP-HF study is a prospective, randomized, controlled trial, which recruited patients with at least one risk factor for ventricular dysfunction, including hypertension, hypercholesterolaemia, obesity, coronary artery disease or diabetes mellitus^22,23^. For this study, we only selected participants without diabetes. Non-diabetic patients from the STOP-HF cohort were confirmed by measuring fasting blood glucose levels. This is part of the usual STOP-HF annual review programme. Patients with readings outside the normal range were referred for follow up. Individual glucose readings are not available for the analysis in this study. Study participant characteristics from both study groups are presented in Table 1. All subjects provided written informed consent to participate in the relevant parent FIELD or STOP-HF study, inclusive of biomarker studies, as per the principles outlined in the Declaration of Helsinki. The STOP-HF study was approved by the research ethics committee of St. Vincent’s University Hospital, Dublin. The FIELD study was approved by the University of Sydney Human Research Ethics Committee. The current analysis was approved as part of both studies.

**Table 1:** Clinical characteristics of control and type 2 diabetes groups.

Clinical characteristics	Control (n = 119)			Type 2 diabetes (n = 234)			*p* value
Age (years)	64 ± 12			62 ± 7			0.23
BMI (kg/m^2^)	28.8 ± 5.4			28.5 ± 5.0			0.59
Male (%)	60			51			0.14
CVD (%)	41			19			**< 0.001**
Smoking category	Yes	No	Ex	Yes	No	Ex	
Smoking (%)	13	46	39	8.1	45.7	45.3	0.92
SBP (mmHg)	135 ± 19			139 ± 19			**0.03**
DBP (mmHg)	83 ± 12			81 ± 11			0.16
Total cholesterol (mmol/L)	4.7 ± 1.0			5.5 ± 1.2			**< 0.001**
HDL-C (mmol/L)	1.38 ± 0.51			1.25 ± 0.33			**0.02**
FKBPL (ng/ml)	1.82 ± 0.79			2.00 ± 0.93			0.07
FKBPL (ng/ml) adjusted for CVD status	1.79 ± 0.89			2.03 ± 0.90			**0.02**

Continuous data are expressed as mean (standard deviation) and categorical data as the number of subjects (%). *p* values < 0.05 (statistically significant). Independent samples t-tests and χ^2^ tests were used as appropriate. HDL-C: High-density lipoprotein cholesterol; SBP: Systolic blood pressure; DBP: Diastolic blood pressure. *p* values <0.05 are in bold.

### FKBPL analysis

Plasma FKBPL concentrations were measured in 364 patients using a validated FKBPL ELISA assay (Cloud-Clone, China) as per manufacturer’s instructions. Samples were stored at − 80 °C until analysis. Average intra- and inter-assay CV for control samples, were 5% and 19%, respectively. Percentages of samples from T2D, non-diabetic control and those with or without CVD loaded onto each plate were kept constant. Plasma samples were analysed in duplicate, and FKBPL concentration for each sample was calculated using a 4-parameter fit based on the standard curve for each plate. Any duplicate samples with CV (%) > 15% were reanalysed and samples were only included in the analysis if the CV was < 15%. For 11 non-diabetic subjects there was inadequate sample volume for reanalysis. Demographics of subjects that we did not include were not significantly different from those who were included (Supplementary Table 1). BNP was measured at the point-of-care in the STOP-HF cohort only as previously reported^21^.

### Statistical analysis

Analysis was performed on 119 Controls and 234 T2D cases. Group comparisons were analysed using independent samples t-tests (for continuous variables) or Mann–Whitney U-test, depending on the normality of the continuous data distribution, or using the χ^2^-test (with Yates’ continuity correction) for categorical variables. Analysis of variance (ANOVA) was used to compare FKBPL levels across 3 smoking categories. Penalised logistic regression was used to derive an efficient model assessing associations of clinical characteristics and FKBPL levels with the presence of clinically evident CVD. The correlation between two continuous variables was assessed based on the Pearson’s correlation coefficient. Statistical significance was defined as *p* < 0.05 (two-sided). Statistical analyses were performed using SPSS software, version 24 (IBM Corp, Armonk, NY, USA).

## Results

### Subject demographics

Study participants without diabetes (n = 119) and with T2D (n = 234) were well matched for age, gender and BMI (Table 1). The percentage of subjects with known CVD was twice as high in the non-diabetic group, however both groups represent only a small subgroup from each of the relevant studies. Smoking status and diastolic blood pressure (DBP) were similar in both groups, whereas systolic blood pressure (SBP) and total cholesterol were higher in patients with T2D. High-density lipoprotein cholesterol (HDL-C) levels were lower in subjects with T2D.

### FKBPL plasma concentration in T2D and/or CVD

There was no difference in FKBPL plasma concentration between those with T2D and non-diabetic subjects. However, after adjustment for CVD status, FKBPL levels were higher in T2D (adjusted mean: 2.03 ng/ml ± 0.90 SD) vs. non-diabetic subjects (adjusted mean: 1.79 ng/ml ± 0.89 SD, *p* = 0.02) (Table 1, Supplementary Fig. 1A). When analysing associations of FKBPL levels with CVD, in a univariate analysis, no differences were observed between subjects with vs. without CVD irrespective of diabetes (without CVD: 1.89 ng/ml ± 0.91 SD vs. CVD: 2.09 ng/ml ± 0.82 SD, *p* = 0.07). However, in the non-diabetic group, plasma FKBPL concentrations were higher in those with than without CVD (CVD: 2.02 ng/ml ± 0.75 SD vs without CVD: 1.68 ng/ml ± 0.79 SD, *p* = 0.02). In diabetes only group, no difference in FKBPL levels was observed between subjects with vs without CVD (control: 1.97 ng/ml ± 0.93 SD vs. CVD: 2.16 ng/ml ± 0.9 SD, *p* = 0.21; Supplementary Table 2).

**Table 2:** Determinants of cardiovascular disease in patients with and without type 2 diabetes—penalised logistic regression coefficients are shown.

Determinants	Control	T2D
BMI	0	0
FKBPL	0.23	0
Age	0.018	0.044
SBP	0.002	0
DBP	0	0
HDL	0	0
Total cholesterol	− 0.360	0
Gender	0.217	0
Smoking	0	0

### FKBPL as a potential diagnostic biomarker of CVD in the absence of diabetes

FKBPL plasma concentration was modestly associated with CVD status in non-diabetic adults, with an area under the curve (AUC) on the receiver operating characteristic (ROC) curve of 0.62 (*p* = 0.02; Supplementary Fig. 1B). When clinical parameters including age, SBP, cholesterol and gender were included in addition to FKBPL, the AUC increased to 0.72 (*p* = 0.01; Supplementary Fig. 1C). Using both diabetes and non-diabetes samples together (n = 351), FKBPL, age, SBP, cholesterol, gender and the presence of diabetes, the AUC was 0.73 (*p* < 0.001; Supplementary Fig. 1D). A well-established biomarker of CVD, BNP^24^, in the non-diabetic group provided an AUC of 0.70 (*p* = 0.001; Supplementary Fig. 2A). FKBPL and BNP were positively correlated (r_s_ = 0.299, *p* = 0.001, n = 119). BNP together with clinical parameters (age, SBP, cholesterol and gender) showed an AUC of 0.77 (*p* = 0.001; Supplementary Fig. 2B). Furthermore, using penalised logistic regression, we identified a number of determinants of CVD in patients without diabetes and only one clinical determinant i.e. age in the T2D group (Table 2). In the non-diabetic group, FKBPL, age, SBP, total cholesterol and gender were associated with CVD (Table 2). Interestingly, total cholesterol was negatively correlated with the incidence of CVD, however, this could be related to the use of statins, considering this was a high-CVD risk non-diabetic group from the STOP-HF trial. Interestingly, FKBPL plasma levels were higher in men compared to women (2.19 ng/ml ± 0.89 SD vs. 1.66 ng/ml ± 0.79 SD, p˂0.001, Supplementary Table 3). Also, a difference in FKBPL levels was observed between smokers (2.43 ng/ml ± 1.05 SD), ex-smokers (2.13 ng/ml ± 0.84 SD) and non-smokers (1.68 ng/ml ± 0.81 SD) in the aggregated groups (ANOVA *p* ˂0.001; Supplementary Table 4).

**Table 3:** Univariate correlations between FKBPL and other clinical parameters in diabetes.

Variable	N	Correlation	
		Coefficient	*p* value
Age (years)	234	0.30	**< 0.001**
Duration of diabetes (years)	234	0.18	**0.006**
Glucose (mmol/L)	233	− 0.14	**0.03**
HbA1c (%)	233	− 0.13	**0.04**
C-peptide (nmol/L)	231	0.18	**0.007**
Urinary ACR	229	0.20	**0.002**
Waist/Hip ratio	234	0.22	**0.001**
SBP (mmHg)	232	0.04	0.544
DBP (mmHg)	232	− 0.14	**0.03**
Triglycerides (mmol/L)	234	− 0.07	0.282
HDL-C (mmol/L)	234	− 0.11	0.099
Total cholesterol (mmol/L)	234	− 0.03	0.96

*p* values <0.05 are in bold.

**Table 4:** Correlations between FKBPL and ECHO variables in non-diabetes/control group.

ECHO variable	N	Correlation	
		Coefficient	*p* value
EF	119	− 0.04	0.68
LA volume	119	0.30	**0.001**
LV mass	119	0.15	0.10
LVIDd	119	0.07	0.44
LVIDs	119	0.06	0.55
IVSd	119	0.18	**0.049**
PWd	119	0.098	0.29
Aortic_root	119	0.11	0.22
Left_atrium size	119	0.27	**0.003**
Peak_E	116	− 0.18	**0.049**
DT (ms)	116	0.33	** < 0.001**
E/A ratio	119	− 0.18	**0.048**

*p* values <0.05 are in bold.

Furthermore, FKBPL demonstrated strong correlations with several clinical characteristics and traditional risk factors in the T2D group. As shown in Table 3, FKBPL was positively correlated with age, known duration of diabetes, C-peptide level, urinary albumin to creatinine ratio (ACR), and waist to hip ratio. Negative correlations were observed between FKBPL and blood glucose, HbA1c, and DBP. In the non-diabetic control group, FKBPL showed correlation with measurements of cardiac structure and function based on a number of echocardiography parameters. Notably, parameters that are important features in diastolic dysfunction were correlated with FKBPL levels, including positive FKBPL correlations with left atrium volume (*p* = 0.001) and size (*p* = 0.003), interventricular septal thickness at end of diastole (IVSd; *p* = 0.049), and deceleration time (DT; *p*  <0.001), and negative correlations with peak E (*p* = 0.049) and E/A ratio (*p* = 0.048; Table 4).

## Discussion

In this cross-sectional study, we investigated the associations between circulating FKBPL in T2D and CVD using age-, BMI- and gender-matched participant samples from two major studies; T2D samples were obtained from the FIELD study^20^ and non-diabetic high CVD risk samples from the STOP-HF study^21^. We demonstrated that FKBPL plasma concentrations were higher in patients with T2D than in controls, when adjusted for the presence of CVD. Furthermore, FKBPL levels were negatively correlated with characteristic metabolic parameters for T2D, glucose and HbA1c, and positively correlated with C-peptide (reflecting endogenous insulin secretion) and the duration of diabetes, in the FIELD study samples. Given that FKBPL is involved in glucocorticoid receptor signalling, and we have demonstrated correlations with glycaemia and insulin secretion, FKBPL may be implicated in the complex mechanisms of glucose control; specifically we observed that better glucose control is associated with high levels of FKBPL^10^. The role of glucocorticoids via glucocorticoid receptors is well established in glucose production and metabolism by stimulating gluconeogenesis, and reducing insulin secretion and glucose uptake^25^. It is possible that this represents a compensatory mechanism in diabetes, which may be mediated e.g. via miRNAs involved in insulin signalling and glucose transport via the PI3K/Akt/m-TOR signalling pathway. Whilst this is speculative, other members of the immunophilin family, which include FKBPL, regulate this pathway and are also implicated in T2D phenotype and associated vascular complications^26^. Inhibition of the PI3K/Akt/mTOR pathway has been shown to lead to cardiomyocyte autophagy triggered by reactive advance glycation end products in diabetes^27^. The mTOR pathway has been shown to regulate insulin secretion and signalling as well as endothelial function thus it is also implicated in cardiovascular complications of diabetes^28^. Nevertheless, this needs to be investigated in future basic science and clinical studies.

In our T2D group, positive correlations were also observed between FKBPL levels and age, urinary ACR, waist/hip ratio whereas FKBPL was negatively correlated with DBP. As age was the only determinant of CVD in the T2D group it is possible that FKBPL is indirectly involved in cardiovascular complications of T2D, which merits further investigation into the FKBPL mechanism rather than its biomarker potential in diabetes. Previously published work on FKBPL demonstrated that FKBPL has a key role in physiological and pathological angiogenesis and that whilst *Fkbpl*^+*/−*^ haploinsufficient mice displayed a pro-angiogenic phenotype, early signs of vascular dysfunction were also observed^15^. There is a plethora of evidence to suggest that in the presence of diabetes, endothelial dysfunction leads to increased vascular permeability and irregular angiogenesis by inducing inflammation, which can lead to atherosclerosis and CVD^29,30^. Nevertheless, in our T2D group there was no difference in FKBPL plasma concentrations between subjects with and without CVD, though low subject numbers may limit statistical power. Interestingly, in the non-diabetic group, the plasma concentration of FKBPL was higher in the presence of CVD. This might suggest that in the presence of diabetes there is a compensatory vascular mechanism, which attenuates this increase in FKBPL levels. In terms of the strength of association of FKBPL level with CVD, in the non-diabetic group, the AUC was comparable to that of an established biomarker for CVD, BNP, when adjusted for important clinical parameters. A similar AUC was observed when both groups were combined together and adjusted for clinical parameters and the presence of diabetes. Previous reports on the biomarker role of BNP in asymptomatic (Stage-B) heart failure demonstrated similar AUC for both diabetic and non-diabetic patients^31^. In the same group, BNP was found to be positively correlated with age, female gender and DBP^32^, whereas, in this study, FKBPL was positively correlated with age and BNP and negatively correlated with DBP in the diabetic group (Table 3). FKBPL plasma concentration in female participants from both groups were lower than male participants (Supplementary Table 3). Previous reports from the STOP-HF trial demonstrated that screening with BNP in primary care and combined with a collaborative care intervention reduced cardiovascular complications, including LV systolic dysfunction, diastolic dysfunction and heart failure^21^. Based on our results and the AUC measurements from the ROC curve, in a subgroup of patients from the STOP-HF trial, both FKBPL and BNP demonstrate similar biomarker potential. Using penalised logistic regression, FKBPL was identified to be associated with CVD only in the non-diabetic patient group together with age, SBP, total cholesterol and gender. These clinical parameters have been associated with CVD in a number of studies previously^33,34^. Furthermore, in the non-diabetic group, FKBPL was correlated with echocardiographic parameters indicative of diastolic dysfunction. Disturbed angiogenesis has been previously implicated in diastolic dysfunction^35^ and in HFpEF^36^. Even though we did not have echocardiogram data available in the diabetic group to explore for correlations with FKBPL, previous reports in diabetic rats demonstrated that increased cardiac angiogenesis is associated with reduced diastolic dysfunction^37^, which could suggest that reduction in FKBPL might be beneficial in these settings.

## Strengths and limitations

This is the first cross-sectional study that shows associations between plasma FKBPL levels and T2D and CVD status using samples from two clinical studies with well-characterised subjects. Study limitations include its cross-sectional nature, and modest sample sizes. In the non-diabetic group, the number of people with CVD was overrepresented in comparison to the diabetic group because this study recruited subjects with CVD risk factors; the selected subgroup excluded people with diabetes. Nevertheless, a strength was that the two groups were well matched in terms of age, BMI and gender. We did not have echocardiographic parameters and BNP measurements for the diabetic group, which limited correlations between FKBPL and echocardiographic parameters and BNP only to non-diabetic group of patients. Whilst the current commercially available FKBPL ELISA kit had modest CVs, each sample was analysed in duplicate on the same plate and only those with a CV under 15% were included. The biomarker potential of FKBPL was investigated in combination with other established clinical risk factors for CVD and the levels correlated with characteristic metabolic and cardiac parameters.

## Conclusions

In this cross-sectional study, we identified associations between the novel anti-angiogenic protein, FKBPL, and T2D parameters and CVD. FKBPL could be involved in the pathogenesis of cardiometabolic diseases including T2D, CVD and cardiac dysfunction. The biomarker role of FKBPL in CVD should be investigated further in larger studies, including cohorts of non-diabetic patients at low-risk for CVD. The therapeutic and diagnostic role of FKBPL in this setting may warrant further investigation, particularly in the absence of diabetes.

## Supplementary information

Supplementary Figures.

Supplementary Tables.

## Data Availability

The datasets used and/or analysed during the current study are available from the corresponding author on reasonable request.

## Abbreviations

DBP: Diastolic blood pressure
SBP: Systolic blood pressure
ACR: Albumin/Creatinine ratio
BNP: B-type natriuretic peptide
T2D: Type 2 diabetes mellitus
CVD: Cardiovascular disease
FKBPL: FK506 binding protein like
BMI: Body mass index
