# Supplementary material for: FKBPL is associated with metabolic parameters and is a novel determinant of cardiovascular disease
Source: Sci Rep. 2020 Dec 10;10:21655. doi: 10.1038/s41598-020-78676-6 (PMC7730138; doi:10.1038/s41598-020-78676-6)
Supplement: Supplementary file 1 — Supplementary Figures. [file 41598_2020_78676_MOESM1_ESM.pdf]

Title: "FKBPL is associated with metabolic parameters and is a novel determinant of cardiovascular disease"

Authors list: Andrzej S Januszewski MD PhD[1], Chris J Watson BSc PhD[2], Vikki O'Neill BSc PhD[3], Kenneth McDonald MD[4,5], Mark Ledwidge PhD[4,5], Tracy Robson PhD[6], Alicia J Jenkins MD PhD[1], Anthony C Keech MD PhD[1] and Lana McClements MPharm PhD[2,7*]

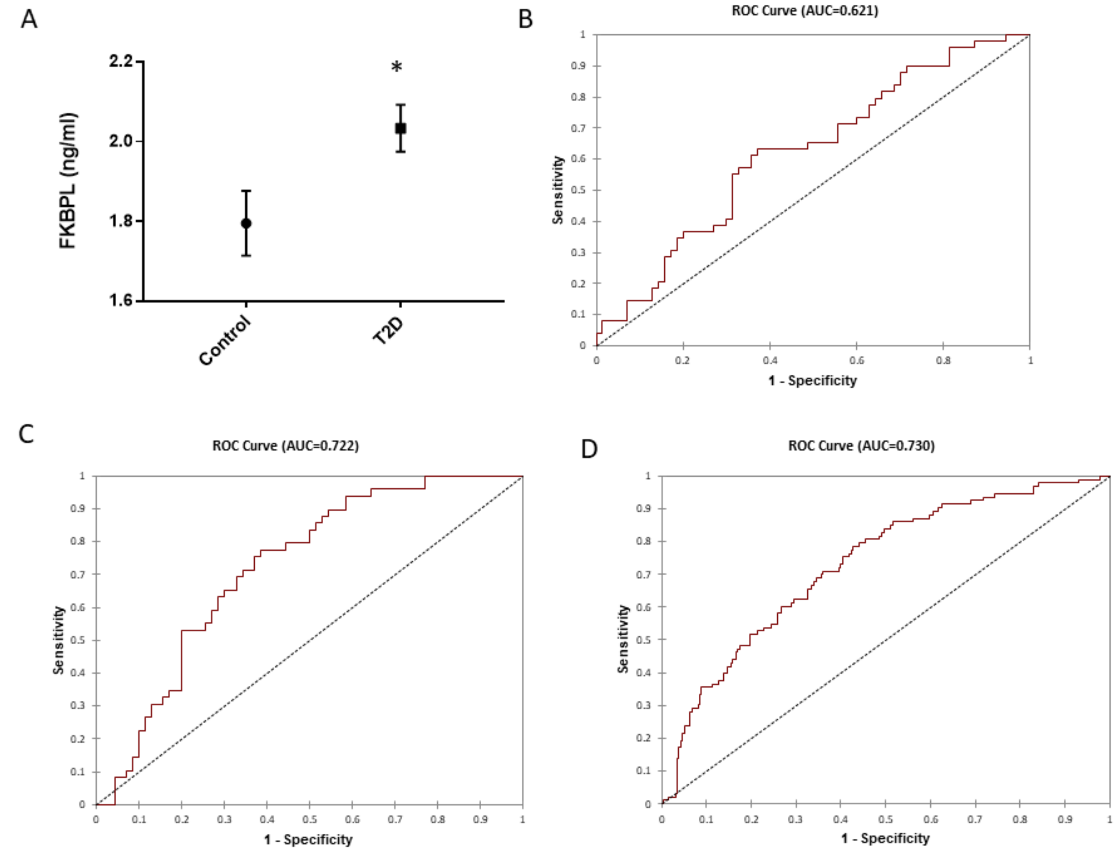

Supplementary Figure 1: Plasma FKBPL concentration is higher in people with type 2 diabetes (T2D) compared to people without T2D, and it is a potential biomarker of the presence of cardiovascular disease (CVD). (A) FKBPL concentration was measured in plasma obtained from participants with (n=234) or without diabetes (n=119) using ELISA. Results were adjusted (least square means) for the presence of cardiovascular disease CVD and compared using F-test. **(B)** Receiver operating characteristic curve of the incidence of CVD in the group without diabetes (n=119). Receiver operating characteristic curve of CVD in the group without T2D based on FKBPL only with area under the curve (AUC) of 0.621 **(C)** and including other clinical characteristics including age, systolic blood pressure (SBP), cholesterol and gender with AUC of 0.722. **(D)** Receiver operating characteristic curve of CVD in both groups combined with and without diabetes (n=351) based on FKBPL, age, SBP, cholesterol, gender and diabetes with AUC of 0.73.

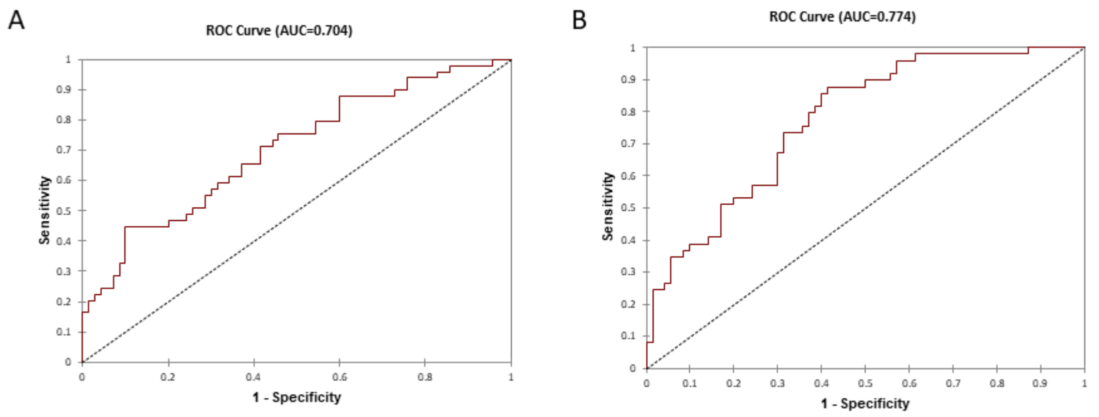

Supplementary Figure 2: BNP as a biomarker of cardiovascular disease (CVD) in the absence of type 2 diabetes (T2D). BNP concentration was determined using ELISA in plasma from patients with and without CVD but in the absence of T2D. (A) Receiver operating characteristic curve of CVD in the group without T2D (n=119) based on BNP only with area under the curve (AUC) of 0.7, and (B) including other clinical parameters such as age, systolic blood pressure (SBP), cholesterol and gender with AUC of 0.774.
