# Supplementary material for: FKBPL is associated with metabolic parameters and is a novel determinant of cardiovascular disease
Source: Sci Rep. 2020 Dec 10;10:21655. doi: 10.1038/s41598-020-78676-6 (PMC7730138; doi:10.1038/s41598-020-78676-6)
Supplement: Supplementary file 2 — Supplementary Tables. [file 41598_2020_78676_MOESM2_ESM.pdf]

Title: "**FKBPL is associated with metabolic parameters and is a novel determinant of cardiovascular disease**"

Authors list: Andrzej S Januszewski MD PhD[1], Chris J Watson BSc PhD[2], Vikki O'Neill BSc PhD[3], Kenneth McDonald MD[4,5], Mark Ledwidge PhD[4,5], Tracy Robson PhD[6], Alicia J Jenkins MD PhD[1], Anthony C Keech MD PhD[1] and Lana McClements MPharm PhD[2,7]*.

Supplementary table 1: Clinical characteristics in non-diabetic group of excluded and included samples

Non-diabetic patients	Excluded (n=11)	Included (n=119)	p-value
Age (years)	61±9	64±12	0.398
BMI (kg/m^2)	29.9±2.6	28.8±5.4	0.51

Supplementary table 2: Univariate analysis of FKBPL levels in patients with versus without cardiovascular disease

	No CVD	CVD	p-value
FKBPL[a]	1.89 ± 0.91	2.09 ± 0.82	0.07
FKBPL[b]	1.68 ± 0.79	2.02 ± 0.75	**0.02**
FKBPL[c]	1.97 ± 0.93	2.16 ± 0.9	0.21

[a]Both groups with and without diabetes combined
[b]Only non-diabetes group
[c]Only diabetes group

Supplementary table 3: FKBPL levels according to gender including both groups with and without diabetes

	Male (n=190)	Female (n=163)	p-value
FKBPL (ng/ml)	2.19 ± 0.89	1.66 ± 0.79	**<0.001**

Supplementary table 4: Univariate analysis of FKBPL plasma levels in different smoking categories

Smoking status	Yes (n=35)	No (n=162)	Ex (n=152)	p-value
FKBPL (ng/ml)	2.43 ± 1.05	1.68 ± 0.81	2.13 ± 0.84	**<0.001**
